# The Present Role and New Potentials of Anaerobic Fungi in Ruminant Nutrition

**DOI:** 10.3390/jof7030200

**Published:** 2021-03-10

**Authors:** Thomas Hartinger, Qendrim Zebeli

**Affiliations:** Institute of Animal Nutrition and Functional Plant Compounds, University of Veterinary Medicine Vienna, 1210 Vienna, Austria; qendrim.zebeli@vetmeduni.ac.at

**Keywords:** additive, anaerobic digestion, cattle, enzyme, anaerobic fungi, herbivore, Neocallimastigomycota, rumen, silage

## Abstract

The ruminal microbiota allows ruminants to utilize fibrous feeds and is in the limelight of ruminant nutrition research for many years. However, the overwhelming majority of investigations have focused on bacteria, whereas anaerobic fungi (AF) have been widely neglected by ruminant nutritionists. Anaerobic fungi are not only crucial fiber degraders but also important nutrient sources for the host. This review summarizes the current findings on AF and, most importantly, discusses their new application potentials in modern ruminant nutrition. Available data suggest AF can be applied as direct-fed microbials to enhance ruminal fiber degradation, which is indeed of interest for high-yielding dairy cows that often show depressed ruminal fibrolysis in response to high-grain feeding. Moreover, these microorganisms have relevance for the nutrient supply and reduction of methane emissions. However, to reach AF-related improvements in ruminal fiber breakdown and animal performance, obstacles in large-scale AF cultivation and applicable administration options need to be overcome. At feedstuff level, silage production may benefit from the application of fungal enzymes that cleave lignocellulosic structures and consequently enable higher energy exploitation from forages in the rumen. Concluding, AF hold several potentials in improving ruminant feeding and future research efforts are called for to harness these potentials.

## 1. Introduction

The metabolic processes in the rumen are of crucial importance in ruminant nutrition and decisively influence the host supply with energy and valuable nutrients. Hence, the rumen microbiota is of key interest in this interdisciplinary research field. Although it is common consensus that the ruminal ecosystem comprises various distinct microbial groups, i.e., bacteria, protozoa, archaea, fungi, and viruses [1], the myriad of microbiota-related studies in ruminant nutrition, however, are predominantly focusing on bacteria. Whilst also the domains archaea and protozoa gained attention in terms of methane emissions or intra-ruminal nitrogen (N) recycling [2,3], AF (phylum Neocallimastigomycota) have yet been widely neglected in the wider ruminant nutrition research. However, these obligate anaerobes are commensals along the gastrointestinal tract of ruminants, being mainly present in the forestomach [4], and truly vital for sufficient fiber degradation by means of expressing various carbohydrate-active enzymes (CAZymes), which can be organized in cellulosomes, as well as physically penetrating plant material via rhizoidal systems [5,6,7].

In fact, AF and their capabilities are highly recognized among microbiologists and comprehensive scientific efforts have been made regarding their taxonomy, lifecycle, and metabolic characterization, which recently was excellently summarized by Hess et al. [5]. Notwithstanding, AF appear to be equally relevant to ruminant nutritionists, who continually seek for strategies to optimize ruminal fiber degradation, a process in which AF are indeed substantially involved [5,7]. Likewise, this microbial clade seems significant for further aspects related to modern livestock feeding. Therefore, by portraying the current perception of AF in ruminant nutrition and outlining their potential in this research field, this review extends the perspective on AF and aims to stimulate scientific discourse on their relevance in modern ruminant feeding as well as sensitize ruminant scientists for these hidden champions in the gut. Since Hess et al. [5] provide an extensive status quo on AF live cycle and taxonomy, the present review will not cover those aspects, albeit a new fungal taxon has again been isolated in the meantime [8]. Similarly, the reader is further encouraged to consult the article of Vinzelj et al. [9] for general basics on AF as well as thorough considerations about their cultivation.

## 2. Current Perception of Anaerobic Fungi in Ruminant Nutrition

In terms of microbiota-related research, the field of ruminant nutrition is doing tremendous efforts to understand how the gut microbiota can be shaped by feeding to optimize rumen fermentation, nutrient provision to the host, as well as gut health and ultimately prevent diseases along with improving animal performance. Thereby, major focus is placed on the rumen, i.e., the gut segment that harbors a highly complex microbial community enabling the energetic utilization of structural carbohydrates as well as providing key nutrients, such as protein and vitamins, to the host animal [1,10]. In the last decades, the hindgut microbiota also became a subject of increasing interest for ruminant nutritionists, particularly in relation to high-grain feeding and associated microbial dysbiosis [11]. With few exceptions and irrespective of gut segment investigated, bacteria represent the target domain in nutritional studies with ruminants, as exemplified by the survey of Henderson et al. [12], who investigated the influence of diet on the ruminal microbiota composition on a global scale, exploring bacteria, archaea, and protozoa. Anaerobic fungi, however, have not been included in this specific study and although AF have been introduced as potent fiber degraders in relevant ruminant nutrition journals very early [7], these microorganisms remain mostly unconsidered so far [12,13,14,15,16]. Therefore, including AF in future ruminant nutrition studies should be the logical outcome, as otherwise, a holistic capture of the rumen microbiota and its implications on nutrition seem hardly feasible. As a complemental note, the authors like to direct additional awareness to AF in the hindgut, which may be more vital for equines at first sight. However, a considerable number of active AF is present in the ruminant’s lower gut [4,17] and since concentrate-rich feeding can shift substrate degradation from the rumen to the hindgut [18], it would be irrational to disregard the fungal population in this part of the gastrointestinal tract.

Interestingly, the rumen simulation technique of Czerkawski and Breckenridge [19] constitutes an in vitro system suitable for long-term incubation that is widespread in ruminant nutrition and may be an excellent option for studying rumen AF in all its aspects. In fact, this system has already been used in the past to explore these microorganisms [20,21,22] and due to its continuous flow of the liquid fraction was actually deemed a better approach for investigating rumen AF than batch cultures [20].

### 2.1. Ruminal Fiber Degradation

It is well established that AF contribute significantly to ruminal fiber degradation by attacking plant cell walls in two ways, i.e., enzymatically, and physically [5,6,7]. Remarkably, recent research showed the affinity of fungal CAZymes for recalcitrant fiber [23], which may explain the particular significance of AF when feeding low-quality forage to ruminants. The authors, however, will not review explicitly on the enzymatic repertoire of AF as fungal CAZymes and the associated cellulosomes have been the subject of earlier reviews [5,24] and a current list of all fungal CAZymes discovered so far is provided by Hess et al. [5].

The synergistic activity of CAZymes, either individually or organized as cellulosomes, and mechanical penetration of plant cell wall by fungal hyphae results in an enhanced cleavage of fibrous structures by AF [5], which will also increase the access for other rumen cellulolytics and likely also proteolytics [2]. Indeed, cell wall degradation by AF can be higher than by bacteria under certain in vitro conditions [25,26] but quantifying the exact fungal contribution to fiber breakdown in the rumen is difficult. However, several in vitro and in vivo studies demonstrated the association between AF and an improved fiber degradability [25,26,27,28], which is also suggested by recent in vitro data from Ma et al. [26]. These authors investigated the impact of co-culturing methanogens with either AF or bacteria on in vitro degradation of rice and wheat straw, observing higher dry matter and fiber degradation in the presence of AF, the extent of which was similar to lignocellulose breakdown by whole ruminal content. Interestingly, acetate production was even higher in the co-occurrence of AF and methanogens than for whole ruminal content or methanogens and bacteria [26], indicating a pivotal involvement of AF in providing this milk fat precursor [29] to the host as also evidenced by others [30] and the generally high acetate formation of AF [31].

Apart from fiber degradation taking place in the rumen, about 5 to 10% of microbial carbohydrate degradation in dairy cows is assigned to the hindgut [11], meaning the microbiota at this intestinal site, and in this way also AF, to be surely relevant regarding total tract fiber degradation in ruminants. As mentioned before, mainly bacteria tended to be routinely studied when investigating the rumen and hindgut microbiota under different feeding regimes [12,13,14,15,16]. Thus, in order to understand better the complex mechanisms of fiber utilization in the gut of ruminants, AF should be added to the list of microbial targets.

### 2.2. High-Grain Feeding

High-grain-induced gut disorders and their implications on animal health constitute one major subject of intensive ruminant production systems [32] and various studies have analyzed the effects on the microbiota in both the rumen and the hindgut [11,32,33,34]. Anaerobic fungi, however, have not been included in the majority of these investigations. Available data predominantly rest upon quantitative real-time PCR and indicate that total fungal abundances in general seem to diminish with increasing starch content of the diet [35,36], which matches the lower ruminal fiber degradation that comes along with such feeding regimes [37]. However, when looking at the fungal composition, a more sophisticated impact on AF by diet becomes apparent. As found for bacteria [34], also the AF richness and diversity in the rumen declines in response to high-grain feeding of dairy cows [36,38,39], which was also observed in the rumen of goats switched from low to high-grain [40]. However, not all taxa are affected negatively [36,41,42] and certain ones indeed proliferate with concentrate-rich feeding, i.e., *Neocallimastix*, *Piromyces*, and *Feramyces* [36,42]. In consequence, these AF could be of specific interest regarding high-yielding dairy cows, which are typically fed high-grain diets during lactation and frequently suffer from impaired ruminal fiber degradation [37]. Thus, complementing AF in future studies on high-grain feeding will provide a more integrated view on alterations in the gut microbiota, which in turn could evince alternative strategies to alleviate gut dysbiosis and its consequences on ruminants. In addition, since parity has recently been recognized as a biological factor that determines the ruminant’s resilience against grain-induced gut dysbiosis and associated health disorders [32,43], it is worth an additional remark that the ruminal AF community seems to be not affected by parity of dairy cows receiving low- or high-grain diets [39].

So far, the investigations on the diversity of fungal community in ruminant nutrition studies were based on sequencing the internal transcribed spacer 1 (ITS1) region, which is still commonly accepted but will likely be outcompeted by the future use of the large 28S rRNA subunit—solely or in combination with ITS1 [5,31,44]. This new barcoding locus shows a similar resolution as ITS1 [45] but is devoid of its drawbacks, such as a high heterogeneity within ITS1 sequence clones [31]. Consequently, prospective ruminant nutrition studies can directly benefit from those refinements in phylogenetic marker candidates and hence improve the knowledge acquisition of coming research.

### 2.3. Emission Reduction

Apart from high-grain feeding, reducing the environmental footprint of dairy and meat production represents a further important subject of ruminant nutrition. Indeed, a sustainable agriculture is of high public interest and in that sense equally relevant to ruminant nutritionists, who insistently pursue to reduce livestock-related emissions, primarily methane, by the use of diverse dietary strategies. Thereby, options such as chemical inhibitors, nitrate, or lipids have been investigated concerning their effect on rumen methanogenesis, fermentation, and microbiota with mainly archaea and protozoa as the main targets of these methane mitigation concepts [3]. Despite this sound strategy with by all means promising outcomes of 20–40% methane reduction [46], AF may constitute a further facet for solving this challenge since methanogens and AF live in a close relationship [47] as demonstrated by substantial cross-feeding of hydrogen and other metabolites [47,48,49]. Likewise, the fungal CAZyme expression and cell wall-degrading activity are increased when culturing AF with methanogens [49,50,51], indicating a beneficial relation between these microbial groups, which is confirmed by higher archaeal methane production from straw when co-cultured with rumen AF than with rumen bacteria [26]. Moreover, methane mitigation of up to 23% by grape seed meal supplementation was earlier associated with decreased total fungal abundance rather than directly with methanogens [22]. Thus, it seems indeed worth to have a closer look at AF regarding their enmeshment in ruminal methanogenesis, particularly during dietary methane mitigation treatments.

In this context of livestock-related emissions, AF have previously attracted the attention of ruminant nutritionists regarding their participation in intra-ruminal N recycling and the associated environmental pollution by N compounds [2]. Certain fungal strains, such as *Neocallimastix frontalis* PNK2, possess high extracellular proteolytic activities [52] and may contribute to an inefficient N utilization by ruminants. In contrast, AF administration enhanced N retention in growing buffaloes [28] and the overall proteolytic capacity of AF seems limited [2,26], meaning a definite statement in this regard seems yet not possible.

### 2.4. Nutrient Source

In addition to the better understanding of their function in the microbial gut community, AF also matter as a protein source for the ruminant. The large majority of crude protein absorbed in the duodenum originates from rumen microbes [53] and AF can represent up to 20% of this microbial biomass [17], thus being surely relevant in terms of amino acid (AA) provision to the host animal. Again, nutritionists have so far focused on microbial protein derived from either rumen bacteria or protozoa [53,54]. In fact, rumen AF possess a highly favorable AA profile and fungal AA were highly digestible for sheep, i.e., showing 90–98% true AA digestibility [55,56]. In spite of such values, extensive research is still needed assessing the actual quantity of fungal protein reaching the small intestine as AF change in abundance under different feeding regimes [36] or may escape sequestration by lasting in the rumen fiber mat.

Apart from constituting a protein source, AF such as *Orpinomyces* sp. are also substantially involved in the ruminal biohydrogenation of linoleic acid [57,58], thus producing conjugated linoleic acids that are absorbed by the host animal. These specific fatty acids can exert diverse health benefits on the ruminant, e.g., a reduced prevalence for hyperketonemia during early lactation as well as modulatory influences on bovine immune cells [59,60], and are further discussed to have anti-carcinogenic effects in humans that consume ruminant-derived products [61]. Consequently, AF significantly improve the nutrient profile for the host, which should be considered when assessing the nutrient supply of ruminants. Whether rumen AF can also synthesize provitamins and vitamins, as has long been known for yeasts and other fungi [62], remains the subject of future research.

### 2.5. Feedstuffs

Microorganisms play a central role in animal feed science, either by being a major part of the feedstuff, e.g., in brewers’ grains, or by their metabolic activity that is capitalized on conserving feedstuffs for ruminants. Silages constitute a main forage source fed to ruminants in all production systems worldwide and microbial additives are frequently applied during ensiling to improve lactic acid fermentation in the silo [63]. Due to the outstanding relevance of silages in ruminant nutrition and the yet untapped potential of AF in this forage conservation method, this field will be elucidated in a separate chapter below.

Likewise, probiotic feed supplements are commonly administered to improve the performance and health of ruminants, particularly since concerns have arisen about the use of antibiotic growth promoters [64]. Supplying exogenous AF to ruminants has been tested sparsely with indeed promising outcomes, and the current applicability of AF as microbial feed additives with its related obstacles will be thoroughly scrutinized in the following chapter.

## 3. Anaerobic Fungi as Feed Additives for Ruminants

Beneficial effects of the probiotic AF strain *Piromyces* sp. FNG5 on ruminal fermentation and performance of buffaloes fed wheat straw and concentrate have been demonstrated by Paul et al. [28]. These authors observed improved ruminal degradation of fiber fractions and organic matter, as well as a higher N retention and increased total tract digestibilities of organic matter and fiber fractions in AF-treated ruminants, which was likely caused by higher activities of fungal cell wall-degrading enzymes [28]. As outlined in Table 1, also others showed enhanced growth and milk performance of calves and lactating buffaloes, respectively, as well as higher ruminal fiber degradability in response to administration of different AF strains [65,66,67,68]. Hereby, it is notable that most of these applied fungal strains were isolated from non-domesticated ruminants, suggesting that wild herbivores may be valuable reservoirs for future probiotic candidates.

Despite such indeed auspicious findings about treatments with AF cultures, the administration form needs to be considered, too, as it is decisive for the practical meaning and feasibility. The AF were introduced into the rumen on a daily or occasionally on a weekly basis as fresh cultures using oral drenching [65,66,67], the rumen cannula [28,68], or by immediately feeding fresh fungal cultures mixed with concentrate [28]. Such laborious approaches are limited to academic purposes only but certainly not implementable in work routines of ruminant livestock production. Due to the resilience of the rumen microbiota against exogenously added microorganisms [69], a one-off drenching dose with AF, however, would not provide a lasting effect and probiotic AF must be administered to the animals continuously. The vast majority of feed additives for ruminants is supplied via the diet and this may constitute the sole easy to apply option for AF-based probiotics, as well. However, fungal cultures are highly susceptible to oxygen and in order to use them as feed additives that need to be storable, further preventing measures are indispensable. In this regard, the initial findings from Paul et al. [70] may be of particular interest, showing that the weekly feeding of *Neocallimastix* sp. CF-17 cells, specifically encapsulated, and hence protected from harmful influences, increased the growth performance of buffalo calves during their first four weeks of life—although, it must be noted that the deployed encapsulation enabled a protection of AF from air for only up to 12 h [70], thus pointing out to the tedious development work still to be done. Apart from encapsulation methods, tenacious AF resting forms that are commonly found in feces and withstanding air exposure and desiccation for several months [4], might hold a further chance for the future that should be pursued. Notwithstanding this, growing fungal cultures on a large scale seems yet not possible [9,71], and besides investigating the suitability of AF encapsulation and fungal resting forms, also advances in cultivation are the prerequisite to enable the full-scale application of probiotic AF in ruminant livestock industry.

Since the development of probiotic AF definitely maintains a long-term challenge, another option for promoting rumen AF by feed additives is implied from yeast-based research data. Indeed, supplementing yeasts to high-grain fed dairy cows alleviated fungal dysbiosis in the rumen as shown by higher AF richness, as well as increased abundances of specific genera, e.g., *Neocallimastix* [38]. Similarly, Chaucheyras et al. [72] have earlier demonstrated a promoting effect by yeasts on the germination and cellulolytic activity of *Neocallimastix frontalis* in vitro. Thus, there seems to be chance that no direct AF-based probiotic is needed, but live yeast preparations that are already common feed additives in ruminant nutrition [64] could shape the AF community beneficially. It is therefore conceivable that ameliorations observed in response to yeast supplementation [64] were partly due to changes in the fungal community, which should be further investigated.

Interestingly, yet to be clarified is the impact of feedstuff particle length, which in general is an important aspect of ruminant nutrition research, decisively determining the fermentative processes and health in the rumen and hindgut [13,73]. Recent in vitro data revealed varying fungal fermentation activities in response to different grass leaf lengths as incubation of *Neocallimastix frontalis* with long particles (4 cm) resulted in 18.4% more gas and higher relative acetate formation compared to its incubation with short particles (0.5 cm) [74]. Although it remains to be solved, whether this in vitro scenario can be transferred to the rumen, it would mean implications on the efficacy of potential AF-based feed additives that could be different with different diet types.

It is worth of notice that AF may not necessarily be applied as direct-fed microbials to be relevant to ruminant nutrition. Apart from their mechanical fiber breakdown, AF exert numerous CAZymes, which can be organized in cellulosomes and that are highly involved in cell wall degradation [5,6,7]. If isolated appropriately, such fungal enzymes could also be convenient to use as feed additives. So far, the administration of fungal enzymes alone exerts no effect on ruminal fermentation or host performance [68], which is likely ascribed to the rapid degradation of the fungal enzymes in the rumen, thus impeding any potential activity. Provided that the resilience of purified fungal enzymes can be increased, they might become a feed additive in ruminant feeding. Chemical structure modifications, as applied to saponins [75], or a protective agent, analogous to the secretory component of immunoglobulin A that conserves this compound from digestion in the gut [76], may increase the resistance of fungal enzymes in the forestomach and could therefore represent a starting point for further research.

## 4. Anaerobic Fungi as Silage Additives

Ensiling aims to preserve fresh forages via rapid lactic acid fermentation with a maximum recovery of dietary energy and highly digestible nutrients [77]. To achieve this, a variety of microorganisms, their isolated enzymes, or manifold combinations are applied as silage additives [63]. Using AF to affect the ensiling process beneficially, however, has been overlooked until now. Instead, fungi are yet predominantly associated with poor silage quality as the presence of yeasts and molds is linked to aerobic deterioration and mycotoxicoses, respectively, both leading to decreased feed intake, reduced performance, and impaired health of the animal [78,79]. Likewise, investigations on fungal communities in differently produced mixed silages were also related to detrimental fungi only [80].

However, silages may indeed be an application area for AF with great potential as they form numerous CAZymes, which further can be arranged as cellulosomes, as well as physically penetrate fibrous structures during their vegetative stage [5,6]. Thus, adding AF at ensiling may lead to an enhanced cleavage of fiber and lignocellulosic biomass, which then would result in an enhanced ruminal fiber degradability. Consequently, plant biomass like straw that is yet widely unexploited in ruminant nutrition could get upvalued and therefore become a more extensively used diet component in ruminant livestock production systems. Such a mode of action was already indicated by two pilot studies investigating the impact of AF inoculation on quality and ruminal degradation of rice straw or whole crop maize silage [81,82]. The plant materials were ensiled with AF strains isolated from herbivore guts, which in both approaches led to an increased ruminal dry matter and fiber degradability during subsequent incubations either in vitro [82] or in situ [81]. Remarkably, compared to the control silages, whole crop maize silages treated with AF also showed an improved silage quality, i.e., higher lactic acid concentrations along with a lower pH and less acetic acid [82], which was also apparent for pH in AF-treated rice straw silages [81].

Although these data provide first evidence of the AF potential as silage additives, it has to be noted that the two studies [81,82] on AF-treated silages were conducted at minor lab-scale. Immense quantities of AF cultures would be necessary to inoculate the large volumes of forage material sufficiently, which are moreover ensiled in very few days on cattle farms. As the production of large amounts of actively growing AF cultures is by now not possible and long-term storage of AF cultures is inevitably associated with substantial viability losses [71], an upscaling to typical silo bunker sizes seems not feasible—particularly when further considering the associated logistical burden for punctually shipping the AF cultures to deployment location without impairments in quality or viability.

Despite this clear limitation in producing AF cultures and in consequence also in using AF as silage inoculants, data of Lee et al. [81] may already implicate a conceivable solution. In fact, fungal populations declined very rapidly after ensiling and the concentrations of thallus-forming units were similar to the control after 60 days [81]. Thus, silages may actually not provide conditions necessary for AF to survive and proliferate, which in turn also precludes a physical penetration of fiber by fungal rhizoids. However, alterations in fiber fraction concentrations in AF-treated silages were observed and still apparent after 60 days of ensiling, such as a further decrease of the neutral detergent fiber content [81], suggesting that not the AF but their secreted CAZymes and cellulosomes were responsible for the differences in chemical composition and the significantly improved ruminal degradability of silages.

**Figure 1:**
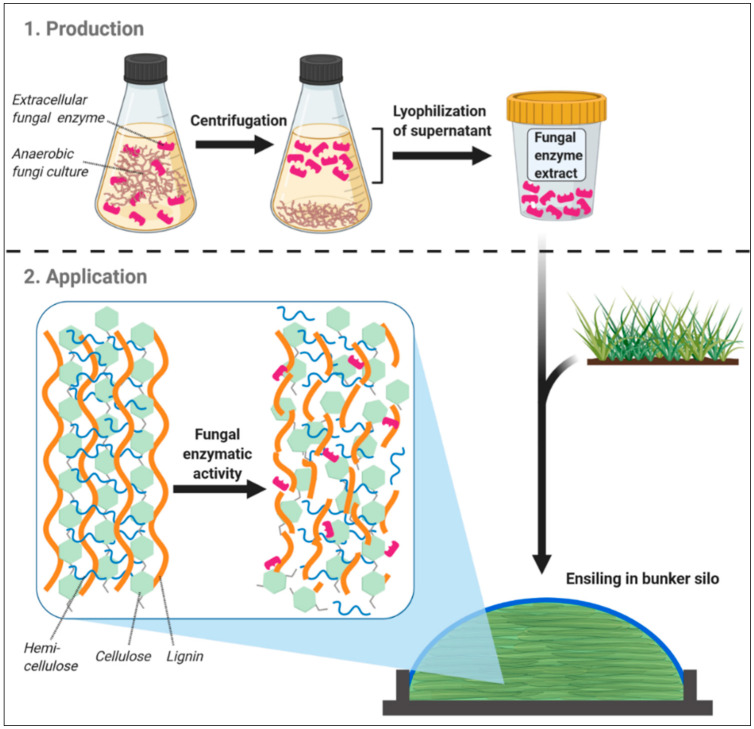
Basic scheme of the production and application of an anaerobic fungi-based silage additive, as well as its potential mode of action during ensiling. Created with BioRender.com.

Consequently, it appears that the enzymatic array of AF can be deployed as silage additives. Indeed, AF-derived enzymes possess several advantageous properties, predestinating them for this purpose: fungal CAZymes are widely expressed extracellularly [24,83] and highly effective in degrading cell wall structures over a wide pH range (pH 4–8) [82], which is further emphasized by the target affinity of AF CAZymes for recalcitrant fiber components [23], i.e., those that are insufficiently degraded in the forestomach system [84]. The latter AF feature is of particular relevance because less robust fiber structures, such as hemicelluloses, are also degraded by lactic acid-induced acidolysis in the silo [85] or else can later be fermented efficiently by rumen microorganisms [84]. Thus, disassembling also recalcitrant fibrous components would mean a more complete cleavage of plant fiber in the silo than it is likely feasible so far, which may be particularly true for strawy biomass, where enzyme additives are assumed to be more effective [63]. Apart from CAZymes, AF form a variety of cellulosomes that can increase the cellulolytic activity immensely [86] and in contrast to bacteria, fungal cellulosomes are not necessarily attached to the cell wall but largely released into the surrounding [24,83]. Regarding the applicability in ruminant nutrition, this would offer the possibility to culture AF and use their spent culture medium (SCM), in which fungal CAZymes and cellulosomes are accumulated [68], as silage additives (Figure 1).

These fungal cultures may then be cultivated in continuous-flow cultures, where AF showed up to 20 times larger production of cellulolytic enzymes than in batch cultures [20]. A lyophilization step may be included in order to facilitate convenient storage and shipping without significantly impairing fungal enzyme activity [87], and might further be combined with other technologies, such as ultrafiltration, to concentrate AF enzymes [88], and therefore potentate their activity as a silage additive. Although growing of AF cultures on a large scale is yet problematic, such production and processing of fungal SCM could be conducted continuously and therefore better enable an upscaling of fungal enzyme application to silo bunker sizes present in ruminant livestock production.

In fact, using AF-derived CAZymes should allow a much more controlled manipulation of the ensiling process than it would be feasible with inoculating viable AF. First, administering AF enzymes precludes the risk of AF metabolizing easily fermentable carbohydrates in favor of fiber due to catabolite repression, which would otherwise mean an unintentional competition for nutrients between AF and lactic acid bacteria. Instead, further substrate from fungal enzyme-induced lignocellulose solubilization may be available for the lactic acid bacteria, which in parts might explain the improved silage quality observed in AF-inoculated silages [81,82]—or else these released nutrients would subsequently be readily accessible to the rumen microbiota, provided they are not metabolized by yeasts after silo opening, thereby promoting aerobic deterioration of silages. Secondly, literature describes a tannin-degrading activity by several AF strains [89], which may also have implications on the outcome of ensiling. Tannins are frequently applied to preserve true protein in silages [90] and tanninolytic AF could counteract this purpose. Combining tannins with AF-derived enzymes, however, would bypass this risk and could yield higher nutritive value of silages, also in terms of protein quality.

## 5. Conclusions

Anaerobic fungi have yet been marginally perceived in ruminant nutrition, particularly when compared to bacteria. However, AF are of outstanding significance for plant cell wall degradation in the rumen—but rather than being relevant for fibrolysis only, AF seem of key importance in other aspects of ruminant nutrition, as well, such as methane formation and nutrient provision to the host. Consequently, it seems prudent and also overdue to consider AF with greater emphasis in prospective ruminant nutrition studies. Indeed, these microorganisms may likely contribute to the clarification of open questions in ruminant feeding that remain unexplained with current knowledge. Regarding the application options in ruminant nutrition, AF may be used as feed additives as well as in silage production. Apart from limitations in the scalability of AF cultivation, advances in AF administration are required to realize higher ruminal fiber degradation as well as improved performance characteristics that have been shown in response to AF treatments of ruminants. Likewise, silage production can become an important application area for AF with their extracellularly produced CAZymes and cellulosomes enabling the successful conservation of forages along with improving their ruminal fiber degradability. Therefore, coming research initiatives should follow up the examination of AF to understand better their role in the gut and how to exploit their application potential optimally, thus shaping tomorrow’s ruminant nutrition.

## Figures and Tables

**Table 1:** Overview of studies investigating anaerobic fungi (AF) as feed additives in ruminants.

AF Strain	Investigated Ruminant Species	AF Administration Form	Results ^1^	Reference
*Piromyces* sp. FNG5 isolated from wild blue bull	Buffaloes fed wheat straw ad libitum with additional concentrate (up to 50% of DM intake)	Fresh fungal culture medium mixed with concentrate before morning feeding, daily administered	Increased total tract digestibility of DM ^2^, organic matter, NDF ^3^, and ADF ^4^ Increased nitrogen retention	[28]
*Piromyces* sp. FNG5 isolated from wild blue bull	Buffaloes fed wheat straw ad libitum with additional concentrate (up to 50% of DM intake)	Fresh fungal culture medium via the rumen cannula, daily administered	Increased DM intake Increased in situ disappearance of NDF and ADF Higher ruminal concentrations of total SCFA^5^, acetate, propionate, butyrate, valerate, iso-butyrate, and iso-valerate Higher abundance of total fungi, large holotrichs, as well as total, cellulolytic, and hemicellulolytic bacteria in the rumen Higher ruminal concentrations of carboxymethyl cellulase, microcrystalline cellulase, xylanase, acetyl esterase, ferulyl esterase, and protease	[28]
*Orpinomyces* sp. C-14 isolated from domesticated cattle	Crossbred calves fed wheat straw and concentrate (50:50 on a DM basis) with additional green oats (1 kg/d)	Fresh fungal culture medium by oral drenching, weekly administered	Increased daily and total body weight gain Increased total tract digestibility of DM, crude protein, NDF, and ADF Lower pH and ammonia nitrogen concentration in the rumen	[65]
*Orpinomyces* sp. C-14 isolated from domesticated cattle	Lactating buffaloes fed wheat straw and concentrate (50:50 on a DM basis) with additional green corn (6 kg/d)	Fresh fungal culture medium by oral drenching, daily administered	Increased milk yield and milk fat content Increased total tract digestibility of DM, organic matter, NDF, and ADF Higher ruminal concentrations of total SCFA, ammonia nitrogen and fungal zoospores	[66]
*Piromyces* sp. WNG-12, isolated from wild blue bull	Lactating buffaloes fed wheat straw and concentrate (50:50 on a DM basis) with additional green corn (6 kg/d)	Fresh fungal culture medium by oral drenching, daily administered	Increased milk yield and milk fat content Increased total tract digestibility of DM, organic matter, crude protein, NDF, and ADF Higher ruminal concentrations of total SCFA, ammonia nitrogen, and fungal zoospores Higher feed efficiency (milk yield in relation to DM intake)	[66]
*Orpinomyces* sp. C-14 isolated from domesticated cattle	Buffalo calves fed wheat straw, concentrate and green oats (45:45:10 on a DM basis)	Fresh fungal culture medium by oral drenching, daily administered	Increased total body weight gain and higher feed conversion ratio Increased total tract digestibility of DM, crude protein, NDF, and ADF Higher ruminal concentrations of total SCFA, ammonia nitrogen, and fungal zoospores, as well as lower ruminal pH	[67]
*Piromyces* sp. WNG-12, isolated from wild blue bull	Buffalo calves fed wheat straw, concentrate and green oats (45:45:10 on a DM basis)	Fresh fungal culture medium by oral drenching, daily administered	Increased total body weight gain and higher feed conversion ratio Increased total tract digestibility of DM, crude protein, NDF, and ADF Higher ruminal concentrations of total SCFA, ammonia nitrogen, and fungal zoospores, as well as lower ruminal pH	[67]
*Orpinomyces* sp. KNGF-2 isolated from Korean native black goat	Sheep fed orchard grass and concentrate (70:30)	Fresh fungal culture medium via the rumen cannula before morning feeding, daily administered	Increased ruminal concentration of total SCFA and acetate, as well as lower pH 3 h post-feeding Increased ruminal concentration of propionate 9 h post-feeding Higher abundances of fungi and bacteria in the rumen Increased enzymatic activity of cellulase and xylanase Increased nitrogen retention Increased total tract digestibility of DM, crude protein, NDF, and ADF	[68]
*Orpinomyces* sp. KNGF-2 isolated from Korean native black goat	Sheep fed orchard grass and concentrate (70:30)	Supernatant of fungal culture medium (i.e., fungal enzymes) via the rumen cannula before morning feeding, daily administered	Increased ruminal concentration of butyrate 3 and 6 h post-feeding	[68]
*Neocallimastix* sp. CF-17 isolated from feces of wild cattle	Buffalo calves fed wheat straw ad libitum with additional 1 kg concentrate and 1 kg green fodder	Encapsulated fungal cells (rhizomycelia and zoospores) mixed with concentrate, weekly administered	Increased body weight gain Increased total tract digestibility of organic matter and NDF Higher ruminal concentrations of total SCFA, carboxymethyl cellulase, and xylanase Higher abundance of fungi, as well as total, cellulolytic, and hemicellulolytic bacteria in the rumen	[70]
*Neocallimastix* sp. CF-17 isolated from feces of wild cattle	Buffalo calves fed wheat straw ad libitum with additional 1 kg concentrate and 1 kg green fodder	Fresh fungal culture medium by oral drenching, weekly administered	Increased total tract digestibility of DM and organic matter Higher ruminal concentrations of total SCFA, carboxymethyl cellulase, and xylanase Higher abundance of fungi, as well as total, cellulolytic, and hemicellulolytic bacteria in the rumen	[70]

^1^ Results observed for anaerobic fungus-treated ruminants in comparison to the control group; ^2^ Dry matter; ^3^ Neutral detergent fiber; ^4^ Acid detergent fiber; ^5^ Short-chain fatty acids.

## Data Availability

Data sharing not applicable.
